# CSF and Plasma Cholinergic Markers in Patients With Cognitive Impairment

**DOI:** 10.3389/fnagi.2021.704583

**Published:** 2021-08-26

**Authors:** Azadeh Karami, Taher Darreh-Shori, Marianne Schultzberg, Maria Eriksdotter

**Affiliations:** ^1^Division of Clinical Geriatrics, Department of Neurobiology, Care Sciences and Society, Center for Alzheimer Research, Karolinska Institutet, Campus Flemingsberg, Stockholm, Sweden; ^2^Division of Neurogeriatrics, Department of Neurobiology, Care Sciences and Society, Center for Alzheimer Research, Karolinska Institutet, Campus Solna, Stockholm, Sweden; ^3^Theme Inflammation and Aging, Karolinska University Hospital, Stockholm, Sweden

**Keywords:** Alzheimer’s disease (AD), choline acetyltransferase (ChAT), acetylcholinesterase (AChE), amyloid beta (Aβ), Cholinergic Index, mild cognitive impairment (MCI), subjective cognitive impairment (SCI), tau protein

## Abstract

**Introduction:**

Alzheimer’s disease (AD) is the most prevalent form of dementia with symptoms of deteriorating cognitive functions and memory loss, partially as a result of a decrease in cholinergic neurotransmission. The disease is incurable and treatment with cholinesterase inhibitors (ChEIs) is symptomatic. Choline acetyltransferase (ChAT), the enzyme that synthesizes acetylcholine (ACh), has been proven recently to be present in both cerebrospinal fluid (CSF) and plasma. As ChAT plays a role in regulating the extracellular ACh levels, it may have an impact on prognosis and cognitive performance in AD patients.

**Objectives:**

To measure ChAT activity and its protein concentration in CSF and plasma from patients with AD, mild cognitive impairment (MCI), or Subjective cognitive impairment (SCI).

**Methods:**

Plasma and CSF samples were obtained from 21 AD, 32 MCI, and 30 SCI patients. The activity and protein levels of ChAT and acetylcholinesterase (AChE), the enzyme catalyzing the hydrolysis of ACh, were analyzed using an integrated activity and protein concentration ELISA-like assay. A Cholinergic Index was calculated as the ratio of ChAT to AChE activities in CSF. The data were analyzed in relation to dementia biomarkers and cognitive performance of the patients.

**Results:**

The CSF ChAT activity was significantly higher (55–67%) in MCI patients compared to AD and SCI cases. The CSF Cholinergic Index was 41 and 22% lower in AD patients than in MCI and SCI subjects, respectively. This index correlated positively with the Aβ_42_/p-tau ratio in CSF in SCI but negatively with that in AD and MCI. The ChAT activity and protein levels in plasma exhibited significant differences with the pattern of AD>>*MCI*>SCI.

**Conclusion:**

This is the first study investigating soluble levels of the key cholinergic enzyme, ChAT, in both plasma and CSF of individuals at different clinical stages of dementia. Although further validation is needed, the overall pattern of the results suggests that in the continuum of AD, the cholinergic signaling exhibits an inverse U-shape dynamic of changes in the brain that greatly differs from the changes observed in the plasma compartment.

## Introduction

Neurodegenerative diseases leading to dementia are common and have major clinical and socioeconomic consequences. According to the World Health Organization (WHO), there are currently about 50 million patients with dementia forecasted to increase to 150 million in 2050 (World Health Organization, 2019). The most common dementia disorder is Alzheimer’s disease (AD), representing about 60%. AD is a progressive and incurable disorder with a successive decline in cognitive functions (Bartus, 2000). Cholinergic neurons in the basal forebrain are particularly liable to the AD-related pathological changes, and the degeneration of these neurons, projecting to the cerebral cortex and hippocampus, is associated with memory problems in AD (Bartus, 2000; Mufson et al., 2008).

About 90 percent of non-excitable cells in the brain are cholinoceptive and sensitive to changes in extracellular acetylcholine (ACh) levels (Vijayaraghavan et al., 2013), the neurotransmitter that is the primary signaling molecule of cholinergic neurons and that stimulates a diverse array of muscarinic and nicotinic receptors. ACh is synthesized by the enzyme choline acetyltransferase (ChAT) in the cytosol of cholinergic nerve terminals, stored in synaptic vesicles and released *via* exocytosis into the synaptic cleft.

In contrast to most other small-molecule neurotransmitters, the post-synaptic activity of ACh is not terminated through its reuptake but by two powerful hydrolytic enzymes, acetylcholinesterase (AChE) and butyrylcholinesterase (BuChE). These enzymes are concentrated in the synaptic cleft, ensuring a rapid decrease in ACh concentration after its release from the pre-synaptic terminal. Under normal conditions, the levels of ACh are regulated through the net result of synthesis by ChAT and degradation by AChE and BuChE to maintain a certain level or duration of action of free ACh in the system. This regulation is suggested to be disturbed in AD through two mechanisms i.e., decreased activity of ChAT, and/or increased activity of AChE and BuChE, both of which lead to a low concentration and reduced turnover of ACh in the cholinergic system (Perry et al., 1978; Atack et al., 1986; Giacobini et al., 1989; DeKosky et al., 1992). Early findings of the loss of cholinergic neurons and marked reduction in ACh in AD led to the subsequent development of the first drugs available for pharmacological treatment of AD, the cholinesterase inhibitors (ChEIs) such as donepezil, rivastigmine and galantamine.

Alzheimer’s disease pathology is also characterized by intracellular neurofibrillary tangles (NFT) and extracellular plaques of aggregated amyloid beta (Aβ) peptide which accumulate in vulnerable brain regions (Sennvik et al., 2000) and both AChE and BuChE have been found aggregated in the amyloid plaques along with Aβ (Darreh-Shori et al., 2009a,b). It has also been shown that, in the presence of excess apolipoprotein E (ApoE), Aβ interacts with these enzymes and forms highly stable BuChE/AChE-Aβ-ApoE complexes (BAβAC), strongly associated with AD pathology (Darreh-Shori et al., 2011a,b, 2012). Formation of these complexes have also been found to act as a switch for the hyperactivation of BuChE and AChE with rapid lowering of ACh levels (Darreh-Shori et al., 2009a,b).

We have reported evidence for the presence of ChAT in human extracellular fluids (Vijayaraghavan et al., 2013) and shown that treatment with galantamine in AD patients resulted in changes in ChAT and AChE in cerebrospinal fluid (CSF) compared with placebo (Karami et al., 2019). Moreover, AD-patients treated with cells releasing nerve growth factor (NGF) in the basal forebrain (Eriksdotter-Jönhagen et al., 2012) also showed changes in the CSF levels of ChAT and AChE (Karami et al., 2015). However, there is still a lack of knowledge on the levels of the key cholinergic enzymes ChAT and AChE in CSF and plasma in untreated patients with AD and in individuals with mild cognitive impairment (MCI) or subjective cognitive impairment (SCI).

The purpose of this study was to investigate possible differences in the cholinergic markers in CSF and plasma from persons with SCI, MCI, or AD, and the relation to cognition and levels of CSF AD biomarkers.

## Materials and Methods

### Subjects

The study population consisted of referrals to the Memory Clinic at Karolinska University Hospital, Huddinge, Sweden, between 2007 and 2009, who were diagnosed with SCI, MCI, or AD. All subjects were thoroughly investigated for memory complaints in the memory clinic. SCI was defined as the presence of cognitive complaints in the absence of pathological neuropsychological testing (Reisberg and Gauthier, 2008; Reisberg et al., 2008). Thus, although the subjects with SCI did not exhibit objective signs of cognitive impairment after extensive clinical and technical work-up and were considered clinically cognitively healthy, they should not be referred to as healthy controls. The AD diagnosis was defined according to the International Classification of Disease 10th revision (ICD-10) criteria (World Health Organization, 1992). The MCI diagnosis was established according to the Winblad criteria (Winblad et al., 2004). The diagnoses for AD, MCI, and SCI were done by a thorough diagnostic workup according to the clinical routine at the Memory clinic, Karolinska University Hospital, including medical history, history from a proxy and clinical examination (in particular, neurological and psychiatric assessments) and cognitive assessment with mini-mental state examination (MMSE). Additionally, evaluation with brain imaging [MRI or computerized tomography (CT)], blood tests and dementia biomarkers in CSF as well as a neuropsychological test battery were performed. The diagnosis was then set at a diagnosis conference with specialists attending.

The inclusion criteria were: (i) lumbar puncture performed as part of the clinical work-up, (ii) diagnosis of AD, MCI, or SCI. Exclusion criteria were: (i) somatic or psychiatric disease significantly affecting cognitive performance, (ii) dementia due to dementia disorders other than AD, and (iii) first-degree relative with AD among the SCI subjects.

All patients and their caregivers provided written informed consent to participate in the study, which was conducted according to the Declaration of Helsinki and subsequent revisions. The regional Ethics Committee in Stockholm County, Sweden approved the study.

### Clinical Assessment and Sample Collection

The subjects underwent a thorough routine work-up at the Memory Clinic, Karolinska University Hospital, Huddinge. Following medical history, history from a proxy and clinical examination including neurological and psychiatric assessment, cognitive screening was performed according to the MMSE test (Folstein et al., 1975). In addition, an extensive cognitive test battery and an MRI scan of the brain were performed.

Plasma samples were collected for routine analyses and cholinergic markers (see below). Lumbar puncture was performed in a standardized manner with the patient in a sitting position between 8 and 12 a.m. to avoid bias by possible circadian fluctuation of CSF biomarkers. The tap was performed with a non-traumatic cannula placed in the intervertebral space L3/L4 or L4/L5. All CSF and plasma samples were aliquoted at the time of collection in polypropylene tubes of 0.5 or 1 ml and were kept stored at −80°C (Darreh-Shori et al., 2004; Vijayaraghavan et al., 2013) until further analysis of the AD biomarkers Aβ42, total tau (t-tau) and phosphorylated tau at threonine 181 (p-tau181, henceforth called p-tau in the main text), and cholinergic markers (see below). CSF and plasma samples were obtained from 21 patients with AD, 32 with MCI, and 30 with SCI.

### Analysis of CSF and Plasma Cholinergic Markers

Choline acetyltransferase activity in CSF samples was analyzed by a colorimetric assay described previously (Vijayaraghavan et al., 2013). A modified version of the assay protocol was used, which sequentially quantified both the enzyme activity and the amount of ChAT protein, as well as the endogenous choline levels. For the activity measurement, undiluted CSF or 400× dilution of plasma samples was used. For measuring ChAT levels in plasma, one of the plasma aliquots was used to prepare the 400× diluted plasma samples. All samples were handled and processed together in the same manner. The dilution buffer for the plasma samples was TBS-TX [10 mM Tris-buffered saline (TBS), pH 7.4, containing 0.05% Triton X-100 and 1.0 mM EDTA].

High-binding 384-well microtiter plates (Nunc MaxiSorp, Denmark) were pre-coated at 4°C with 75 μL/well of a ChAT antibody (MAB 3447; R&D System; reconstituted at a concentration of 250 μg/mL) and incubation overnight. The working solution of this antibody was prepared at 1 μg/mL in carbonate coating buffer, pH 9.0. After the incubation, the plate was washed once with 100 μL/well TBS buffer (10 mM, pH 7.4). Then the antibody-coated wells were incubated with 10 μL/well in triplicates of the samples or a 2-fold serial dilution of a ChAT protein-calibrated pooled plasma standards (ranging from 1.2 to 0.019 μg/mL, prepared in TBS-TX. On a set of wells on the plate that were not coated with the primary antibody, 50 μL/well of a 2-fold serial dilution of choline chloride standards (ranging from 50 to 0.78 μM in TBS-TX) were added. All samples and the ChAT-calibrated plasma standards were applied both as native (unmodified) samples and as denatured (by heating in a thermal cycler 3 × 8 min at 98°C). The denatured samples served as controls for both the ChAT activity and the endogenous concentration of choline in the samples. Then, to all wells, except for the choline standards, 40 μL/well of Cocktail A was added and incubated with for 2 h at 38°C, under constant gentle shaking. The Cocktail A was prepared in TBS-TX, containing 62.5 μM of acetyl-coenzyme A (ACoA), 1.25 U/ml of phosphotransacetylase, 8.75 mM of lithium potassium acetyl-phosphate, 6.25 μM choline chloride and 0.75 mM of eserine hemisulfate. After the incubation, the plate was placed on ice for ∼2 min and centrifuged at 1500 rpm for 1 min to recover the condensed volume. Then the sealing tape was removed and 25 μL/well of Cocktail B was added to all wells including those with choline standards. The Cocktail B was prepared in 50 mM K^+^ Na^+^ phosphate buffer, pH 7.6, containing 0.93 U/ml of choline oxidase, 1/5000 Streptavidin-HRP, 3.0 mM 4-aminoantipyrine and 6.3 mM phenol. The plate was immediately placed in a microplate spectrophotometer reader (Tecan Infinite M1000) and changes in absorbance at 500 nm was monitored at 3 min intervals, enabling assessment of choline concentration using the choline standards.

Choline acetyltransferase activity (nmol/min/mL samples) was calculated based on the difference between the choline concentration (in pmol) in the denatured and native samples, divided by incubation time (in min) at 38°C and volume (in mL) of the samples.

Following reading, the plate was sealed and incubated at 4°C overnight. The plate was then emptied and washed once for 5 min with TBS, then blocked with 100 μL/well of blocking solution (the coating buffer, containing 5% w/v bovine serum albumin) for 1–2 h at room temperature (RT) under gentle orbital shaking. After 3 × 5 min washing with TBS-T (TBS, containing 0.05% Tween 20), the plate was incubated first for 60 min at 38°C with 50 μL/well of detecting antibody solution (anti-ChAT rabbit polyclonal antibody, PAB14536, Abnova, diluted 1/3500). Subsequently, 25 μL/well of a secondary antibody solution (alkaline phosphatase-conjugated polyclonal Swine anti-rabbit antibody, Dako, diluted 1/1700) was added to the wells and incubated for an additional 30 min. Both antibody solutions were prepared in TBS-T, containing 1% BSA and 0.01%NaN_3_. After washing and addition of a substrate solution (10 mM di-sodium p-nitrophenyl phosphate in 1.0 M diethanolamine buffer, pH 9.8, containing 1 M MgCl and 0.01% NaN_3_), the reaction was monitored at 405 nm wavelength using an Infinite^®^ M1000 Tecan microplate reader (Vijayaraghavan et al., 2013).

The activity of AChE was assessed by the modified Ellman’s colorimetric assay described in Darreh-Shori et al. (2008). The corresponding levels of AChE protein in the samples were measured by integrating the activity assay with functional ELISA setup, which measures the protein levels of the functionally intact enzyme, as described in Darreh-Shori et al. (2008). The activity and protein levels of AChE were determined in 5× pre-diluted (in TBS-TX) samples.

### CSF *Cholinergic Index* and the Functional Activities of Cholinergic Markers in CSF and Plasma

To estimate the functional ChAT activity in CSF (ChAT_F_), the overall ChAT activity (in pmol/min/mL) was divided to its protein in CSF (in ng/mL). A similar calculation was used to estimate the functional activity of CSF AChE (AChE_F_). The recently reported *Cholinergic Index* in CSF, obtained by dividing the functional levels of CSF ChAT (in pmol/min/μg) to CSF AChE (in nmol/min/μg) (Karami et al., 2019). Thus, an increase in this *Cholinergic Index* (ChAT_F_/AChE_F_ activities) indicates a net increase in CSF ACh levels.

### Analysis of AD Biomarkers

Analysis of Aβ_42_ peptide, t-tau and p-tau in CSF samples was performed with xMAP technology using the Inno-Bia AlzBio 3 kit (Innogenetics, Gent, Belgium) as described previously (Olsson et al., 2005). We also calculated the CSF ratio Aβ_42_/p-tau, which is a commonly used ratio in AD diagnostic work-up (Duits et al., 2014; Lehmann et al., 2015; Palmqvist et al., 2015; Marizzoni et al., 2019).

### Statistical Analysis

Analysis of variance (ANOVA) test was used to evaluate the differences between the groups when the data fulfilled the normal distribution criteria. In ambiguous cases, the data were transformed using natural logarithm. The significant level was set to *p* < 0.05. A significant ANOVA was followed by Fisher’s PLSD *post hoc* test. Data in the form of ratios or percentages were analyzed with a non-parametric test (Kruskal–Wallis instead of ANOVA), followed by Mann–Whitney *U* test as *post hoc* analysis. Correlation analyses were performed using Pearson correlation analysis and were visualized using simple regression plots. When deemed necessary the correlations were validated using the non-parametric Spearman rank correlation test. All raw data are presented in the table as mean ± SD values, whereas in the text and figures the data are presented as mean ± S.E.M.

## Results

### Demographic and Clinical Characteristics at Baseline

In total, samples and data from 83 (47 women and 36 men) patients with memory impairment were included with a mean age of 71 (±1.1) years and a mean MMSE total score of 26.9 (±0.3) with scores between 17 and 30. The demographic data and levels of cholinergic and AD biomarkers in CSF and plasma are shown in Table 1.

**TABLE 1:** Demographic data overview and measured levels of relevant biomarkers in CSF and plasma.

	***AD***	***MCI***	***SCI***	**Total**
N (women/men)	21 (7/14)	32 (19/13)	30 (21/9)	83 (47/36)
Age in years	77 ± 10	73 ± 9	66 ± 8	72 ± 10
MMSE score	24 ± 3	27 ± 3	29 ± 2	27 ± 3
CSF Aβ_42_ (pg/ml)	519 ± 186	665 ± 298	900 ± 274	713 ± 303
CSF p-tau (pg/ml)	107 ± 43	59 ± 22	49 ± 18	68 ± 36
CSF t-tau (pg/ml)	712 ± 371	316 ± 140	213 ± 79	379 ± 289
Aβ_42_/p-tau181	6 ± 5	14 ± 9	22 ± 12	15 ± 11
CSF AChE activity (nmol/min/ml)	12 ± 4	10 ± 4	10 ± 4	11 ± 4
CSF AChE protein (ng/ml)	48 ± 21	46 ± 24	53 ± 27	49 ± 24
CSF ChAT activity (nmol/min/ml)	1.5 ± 1.1	2.2 ± 1.2	1.3 ± 0.8	1.7 ± 1.1
CSF ChAT protein concentration (μg/ml)	1.0 ± 0.8	0.8 ± 0.3	0.8 ± 0.3	0.9 ± 0.5
Plasma ChAT activity (nmol/min/ml)	117 ± 60	82 ± 81	62 ± 63	83 ± 73
Plasma ChAT protein concentration (μg/ml)	150 ± 83	64 ± 45	44 ± 24	75 ± 63

*The values are means ± standard deviation. Aβ_42_, beta-amyloid peptide 1–42; AChE, acetylcholinesterase; AD, Alzheimer’s disease; ChAT, choline acetyltransferase; CSF, cerebrospinal fluid; MCI, mild cognitive impairment; MMSE, mini-mental state examination; p-tau181, phosphorylated tau 181; SCI, subjective cognitive impairment; t-tau, total tau.*

### CSF ChAT

**FIGURE 1:**
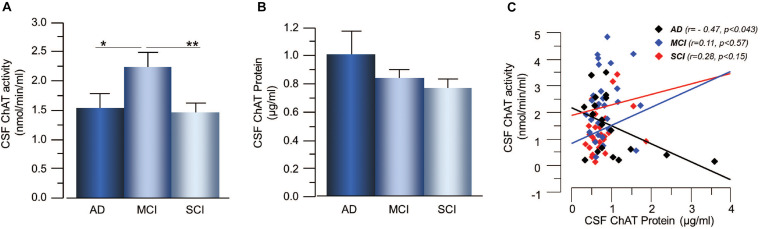
Changes in ChAT levels in CSF of patients in different stages of dementia. **(A)** CSF ChAT activity was higher in patients with the clinical diagnosis of MCI than in AD or SCI. **(B)** The corresponding ChAT protein concentrations in CSF did not show any significant differences. **(C)** The activity and protein concentration of ChAT in CSF exhibited negative correlation mainly in the AD group. AChE = acetylcholinesterase; AD = Alzheimer’s disease; ChAT = choline acetyltransferase; CSF = cerebrospinal fluid; MCI = mild cognitive impairment; SCI = subjective cognitive impairment. *p < 0.05, **p < 0.01.

The activity of ChAT in CSF was significantly higher in the MCI group, i.e., 55 ± 17% higher than in the AD group (*p* < 0.039) and 67 ± 11% higher than the SCI group (*p* < 0.0016, Figure 1A). The CSF levels of ChAT protein did not show any significant differences between the groups (Figure 1B). The CSF ChAT_F_ (i.e., ratio between ChAT activity and ChAT protein concentration in CSF) was significantly higher in MCI patients compared to SCI subjects (53 ± 11%, *p* < 0.0083). There was a significant negative correlation between CSF ChAT activity and its protein concentration in the AD group (*r* = −0.47, *p* < 0.043, Figure 1C), but not among the other groups or the overall subjects.

### Plasma ChAT

**FIGURE 2:**
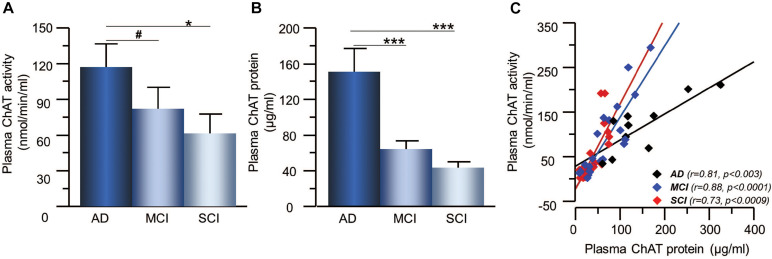
Changes in ChAT levels in plasma of patients in different stages of dementia. **(A)** The plasma ChAT activity was higher in AD compared to patients with a clinical diagnosis of MCI or SCI. **(B)** A similar pattern of differences was found with regard to ChAT protein levels in plasma in the three groups. **(C)** Plasma ChAT activity correlated with its protein concentration in all three groups. AD = Alzheimer’s disease; ChAT = choline acetyltransferase; MCI = mild cognitive impairment; SCI = subjective cognitive impairment. *p < 0.05, ***p < 0.001, ^#^*p* < 0.07.

The plasma ChAT activity was higher in the AD patients, i.e., 57 ± 28% higher than the MCI-group (*p* < 0.071) and 90 ± 26% higher than the SCI-group (*p* < 0.024, Figure 2A). The plasma ChAT protein concentration was significantly higher in the AD patients, i.e., by 198 ± 22% compared to the MCI-group (*p* < 0.0006) and 245 ± 14% higher than the SCI-group (*p* < 0.0001, Figure 2B). The ChAT_F_ in plasma did not differ significantly between the groups. The plasma ChAT activity was positively correlated to the ChAT protein concentration in all groups (*r* = 0.72, *p* < 0.0001) as well as within each group (*p* < 0.003 for AD, *p* < 0.0001 for MCI and *p* < 0.0009 for SCI, Figure 2C). No correlation was found between CSF ChAT activity or protein levels and the plasma ChAT activity (*p* < 0.88) or protein levels (*p* < 0.49).

### CSF AChE

**FIGURE 3:**
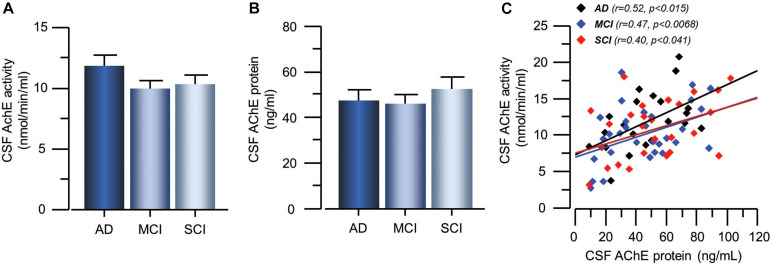
Activity and protein levels of AChE in CSF of the patients. **(A)** The CSF AChE activity did not differ between patients with the clinical diagnoses of AD, MCI, or SCI. **(B)** Similarly, the CSF protein levels of AChE did not differ between the groups. **(C)** The overall AChE activity correlated with its protein levels in the CSF. AChE = acetylcholinesterase; AD = Alzheimer’s disease; CSF = cerebrospinal fluid; MCI = mild cognitive impairment; SCI = subjective cognitive impairment.

The activity and protein concentration of the ACh-degrading enzyme, AChE, in CSF did not show any significant differences between the groups (Figures 3A,B). In order to estimate changes in the level of functional AChE activity (AChE_F_), the ratio between AChE activity to AChE protein levels in CSF were calculated. The AChE_F_ in CSF did not differ between the groups. There was significant correlation between the AChE activity and protein concentration in CSF in all groups (*r* = 0.46, *p* < 0.0001) as well as within each group (Figure 3C).

### The CSF Cholinergic Index

**FIGURE 4:**
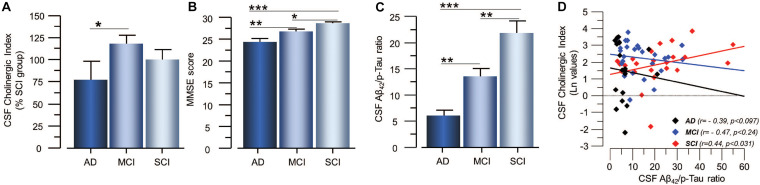
Estimated *Cholinergic Index* in CSF of patients in different stages of dementia and its relation to cognition and the CSF Aβ_42_/p-tau ratio. **(A)** The estimated CSF Cholinergic index (ChAT_F_/AChE_F_) was highest in the MCI group. **(B)** The global cognitive performance of the patients as assessed by MMSE test shows the expected pattern. **(C)** The CSF Aβ_42_/p-tau ratio also follows the expected pattern of lowest in AD and highest in SCI. **(D)** The CSF Cholinergic index showed differential correlation with the CSF Aβ_42_/p-tau ratio in SCI and AD. In panel **(D)**, the CSF Cholinergic index is given in natural logarithm (Ln). The CSF Cholinergic index is defined as a ratio between the functional levels of ChAT (in nmol/min/μg) and the functional levels of AChE (in nmol/min/μg) in CSF (i.e., ChAT_F_/AChE_F_ activities). AChE = acetylcholinesterase; AD = Alzheimer’s disease; ChAT = choline acetyltransferase; CSF = cerebrospinal fluid; MCI = mild cognitive impairment; p-tau = phosphorylated tau 181. SCI = subjective cognitive impairment. *p < 0.05, **p < 0.01, and ***p < 0.001.

Cholinergic signaling is most likely regulated through a balance between synthesis, release and degradation of ACh. We hence defined a CSF *Cholinergic Index* as the ratio between the functional ChAT (in nmol/min/μg) and that of functional AChE (in nmol/min/μg) in CSF (ChAT_F_/AChE_F_). Thus, an increase in this ratio means a net increase in ACh levels. The CSF Cholinergic Index was significantly higher in the MCI group than in the AD patients (41 ± 9% *p* < 0.048, Figure 4A), but not compared to the SCI group (19 ± 9% *p* < 0.21). The Cholinergic Index did not show a statistically significant difference but was just numerically higher in SCI compared to AD (22 ± 12% *p* < 0.32).

### Correlations to Cognition

As expected, lower MMSE scores as well as lower levels of Aβ_42_ and higher levels of t-tau and p-tau in the CSF were found in patients with AD as compared to patients with MCI and SCI (Figure 4B). The CSF Aβ_42_/p-tau ratio was significantly lower in AD patients compared to MCI (34 ± 5%, *p* < 0.001) and SCI (72 ± 10%, *p* < 0.0001), and in MCI patients compared to SCI subjects (38 ± 7%, *p* < 0.0032, Figure 4C). There was a positive correlation between MMSE test scores and the CSF Aβ_42_/p-tau ratio for all cases (*r* = 0.31 *p* < 0.005).

Furthermore, MMSE scores correlated negatively to the levels of t-tau (*r* = −0.39, *p* < 0.0002) and p-tau (*r* = −0.40, *p* < 0.0001) and positively to the levels of Aβ_42_ (*r* = 0.23, *p* < 0.04) in CSF as analyzed in all subjects together (no correlations were found within each group).

Analysis of the correlation between cognition and the CSF Cholinergic Index showed no significant correlation.

**FIGURE 5:**
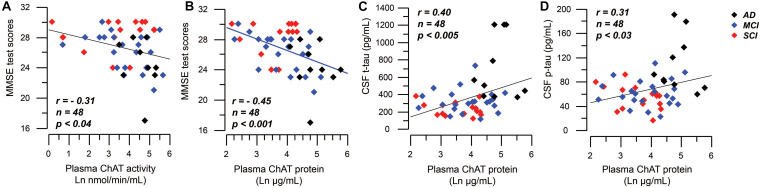
Correlations between plasma ChAT levels and global cognition and CSF tau levels. **(A)** Illustrates a negative significant correlation between global cognitive performance as assessed by MMSE test and the activity of ChAT in plasma. Panel **(B)** shows the corresponding negative significant correlation between MMSE test scores and the plasma ChAT protein. Panels **(C,D)** show the positive correlations between the plasma ChAT protein and the t-tau **(C)** and p-tau **(D)** levels in CSF of all subjects. The plasma ChAT activity and protein levels are given in natural logarithm (Ln). AD = Alzheimer’s disease; ChAT = choline acetyltransferase; CSF = cerebrospinal fluid; MCI = mild cognitive impairment; p-tau = phosphorylated tau181 protein; SCI = subjective cognitive impairment; t-tau = total tau protein.

Mini-mental state examination scores correlated negatively to the plasma ChAT activity (*r* = −0.31, *n* = 48, *p* < 0.04, Figure 5A) and to the plasma ChAT protein for all cases (*r* = −0.45, *n* = 48, *p* < 0.001, Figure 5B). Subgroup analyses indicated that the correlation remained significant only among the MCI patients (*r* = −0.53, *n* = 22, *p* < 0.009).

### Correlations Between Cholinergic Markers and AD Biomarkers

The CSF Cholinergic Index correlated positively with the Aβ_42_/p-tau ratio in the SCI group (*r* = 0.44, *p* < 0.031, Figure 4D), but negatively with the Aβ_42_/p-tau ratio in the AD group (*r* = −0.39, *p* < 0.097).

The CSF Cholinergic Index correlated negatively with the p-tau levels in SCI group (*r* = −0.50, *p* < 0.015), but not in the AD (*r* = 0.44, *p* < 0.056) or MCI group (*r* = 0.17, *p* < 0.40). The CSF Cholinergic Index did not correlate with the t-tau levels in any of the groups.

The plasma ChAT protein correlated positively with t-tau (*r* = 0.40, *n* = 48, *p* < 0.005, Figure 5C) and p-tau (*r* = 0.31, *n* = 48, *p* < 0.03, Figure 5D), but not with the CSF Aβ_42_ level as analyzed in all subjects together. Plasma ChAT activity did not correlate with these markers and no correlations were found within the subgroups.

Correlations were also found between the CSF AChE activity and the levels of CSF t-tau (*r* = 0.28, *p* < 0.011 in all patients, and *r* = 0.40, *p* < 0.03 in the SCI group) and CSF p-tau (*r* = 0.32, *p* < 0.0033 in all patients). The CSF AChE activity also correlated with the levels of CSF Aβ_42_, but only in the MCI group (*r* = 0.38, *p* < 0.032).

### Multivariate Logistic Regression Analysis of Cholinergic and AD Biomarkers

To explore the relationship between cholinergic markers and the AD biomarkers, an exploratory analysis was done on subgroups which were positive or negative with regards to CSF levels of the AD biomarkers: Aβ_42_+ vs. Aβ_42_− (cut-off 650 pg/ml), t-tau+ vs. t-tau− (cut-off 400 pg/mL), or p-tau+ vs. p-tau− (cut-off 78 pg/mL). The results are presented in Supplementary Figures 1, 2. Briefly these analyses indicated that CSF ChAT activity (Supplementary Figure 1) and the CSF Cholinergic index (Supplementary Figure 2) in Aβ_42_+, t-tau+, or p-tau+ groups had very similar pattern to that observed in Figures 1, 4, respectively. This pattern was lost in Aβ_42_−, t-tau−, or p-tau− groups indicating that these cholinergic markers were sensitive to the underlying pathological events in AD.

To further explore the observations, multivariate logistic regression analysis was performed using diagnosis (SCI/MCI/AD) as the dependent variable and the cholinergic enzymes (activity and protein) levels and MMSE test score as the independent variables. This model predicted the diagnosis with 76, 61, and 53% accuracy, respectively. A model that included only MMSE test score and the AD CSF biomarkers (Aβ_42_, t-tau, and p-tau) but not the cholinergic markers had a predictability of 70, 72, and 71% for the diagnoses of SCI, MCI, and AD, respectively.

When the CSF levels of ChAT and AChE were included together with only MMSE and t-tau, the predictability of the model for SCI, MCI, and AD was 84, 75, and 79%, respectively, which was superior to all models lacking the CSF cholinergic variables.

We also performed logistic regression analysis of the plasma ChAT activity and protein levels. A model that included the CSF AD biomarker (Aβ_42_, t-tau, and p-tau) and the plasma ChAT levels had a predictability of 88, 87, and 90% for the diagnoses of SCI, MCI, and AD, respectively. Without the plasma ChAT levels, the corresponding predictability was 77, 66, and 57%. Additional inclusion of the MMSE test score did not improve the predictability of the model that included plasma ChAT levels (69, 82, and 90%, respectively).

## Discussion

The cholinergic hypothesis proposed the importance of cholinergic neuron degeneration in AD and that it was associated with cognitive impairment (Bartus et al., 1982). Since then, many studies have shown that the degeneration of the central cholinergic system is associated with cognitive decline (Drachman and Leavitt, 1974; Davies and Maloney, 1976; Mufson et al., 2008; Ballinger et al., 2016). However, less is known regarding the levels of the ACh-synthesizing enzyme ChAT and the ACh-degrading enzyme AChE in CSF and plasma in patients with cognitive impairment. The aim of the present study was to investigate these enzymes with regard to both their activity and protein level in relation to cognitive function and AD biomarkers. Comparison of data obtained from analysis of samples from patients with AD and MCI as well as individuals with SCI, showed significant differences between the groups and between the patterns in CSF and plasma.

The ChAT activity in CSF was higher in MCI patients compared to AD and SCI. This is in line with studies on the levels of ChAT activity in *postmortem* brain tissue (DeKosky et al., 2002; Ikonomovic et al., 2005). Thus, a difference in ChAT levels in CSF samples may serve to distinguish between clinical and pre-clinical forms of the disease. We have previously hypothesized that CSF ChAT activity and thereby extracellular ACh may be involved in the regulation of astroglial function in the brain (Malmsten et al., 2014). Furthermore, we found that astrocyte activity in the brain may follow an inverse U-shape dynamic in the continuum of AD, with a peak at the MCI stage of the disease (Vijayaraghavan et al., 2013). Given that astrocytes constitute one of the sources of soluble ChAT in CSF (Vijayaraghavan et al., 2013; Darreh-Shori et al., 2014), the present finding that CSF ChAT levels were highest in the MCI group may suggest that CSF ChAT levels reflect an ongoing regulatory process, attempting to modulate a (protective or detrimental) functional status of astrocytes in the brain.

In two independent treatment studies in AD patients (one with encapsulated NGF-releasing cells and the other with a ChEI), we showed that CSF ChAT levels increased in response to the treatment and that the increase was positively correlated with measures of cognition (Karami et al., 2015, 2019). Three months treatment with the ChEI galantamine resulted in increased levels of ChAT activity in CSF (Karami et al., 2019). Encapsulated cells releasing NGF to the basal forebrain in ten AD patients with ongoing treatment with ChEIs resulted in increased levels of ChAT activity in CSF (Karami et al., 2015; Eyjolfsdottir et al., 2016). Thus, higher levels of ChAT activity in the CSF of MCI patients compared to AD patients would suggest that increased activation of extracellular ACh signaling is part of an intrinsic protective rather than detrimental mechanism, which when it fails to cope with the underlying disease processes, results in progress of MCI to AD.

We found a negative correlation between ChAT activity and ChAT protein levels in CSF samples from AD patients, but not in MCI and SCI. Reports indicate that ChAT activity is biologically and pathologically affected in the brain in several ways. One is phosphorylation and dephosphorylation by kinases and phosphatases, which increases and respectively decreases ChAT activity (Dobransky et al., 2000). Another is inhibition by some endogenous molecules which seem to be accumulated in the AD brain (Andriamampandry and Kanfer, 1993). Unpublished data in our laboratory further evince that ChAT is extremely sensitive to oxidative environment, rendering ChAT protein inactive which can be prevented or reversed by a reducing agent such as mercaptoethanol. Overall, the observed negative correlation between the enzyme activity and protein levels of ChAT may suggest that higher ChAT protein levels in CSF of AD patients is a sign of an unsuccessful compensatory mechanism to recover the reduced ACh in synapses, and/or to dampen hyperactivity of astroglial cells (Darreh-Shori et al., 2013; Vijayaraghavan et al., 2013).

In plasma, both the activity and protein levels of ChAT differed between AD, MCI, and SCI patients, according to the pattern of AD>>*MCI*>SCI. This pattern between the groups was completely different from that seen in CSF and may indicate that extracellular cholinergic signaling in the periphery may increase during the disease progression of AD. In addition, both activity and protein levels of ChAT in plasma correlated negatively with global cognition as assessed by the MMSE test. Plasma levels of ChAT protein also correlated with the CSF t-tau and p-tau levels. Indeed, multivariate logistic regression analysis indicated that a model, which included plasma ChAT activity and protein levels had superior diagnostic predictability than a model that included only CSF AD biomarkers (88% vs. 71% overall correct classification). Overall, the pattern of these observations suggested that elevated plasma levels of ChAT protein were related to a more severe stage of the disease as could be deduced by the global cognitive performance and levels of the AD biomarkers, Aβ_42_, t-tau, and p-tau in CSF.

Many studies report the involvement of neuroinflammation and its fundamental role in the progression of the neuropathological changes that are observed in AD. There is ample evidence for the presence of immune-related proteins and cells within close proximity to amyloid plaques in AD (Rogers et al., 1988; Griffin et al., 1989). ACh is important for peripheral functions and cholinergic signals also regulate innate and adaptive immune functions (Shaked et al., 2009; Rosas-Ballina et al., 2011). It has been shown that ACh suppresses or modulates the immune responses to inflammatory cues (Rosas-Ballina and Tracey, 2009). We have shown that, in addition to lymphocytes, both astrocytes and human embryonic stem cells can express and release ChAT into the culture medium in response to inflammatory stimuli (Vijayaraghavan et al., 2013; Malmsten et al., 2014). Given that AD is developed gradually within a time span of 2–3 decades, the observed pattern of ChAT levels in plasma may indicate that at least some of the driving factors of AD originate in the periphery rather than in the brain itself. This is particularly appreciated from the finding that ChAT levels in CSF did not differ between AD and SCI groups. Indeed, in a previous report we showed a close cross-talk between the cytokine levels in plasma and CSF as a function of plasma and CSF BuChE activity, but not AChE activity (Darreh-Shori et al., 2013). In plasma, the levels of soluble ChAT and BuChE are much higher than the levels of AChE (Vijayaraghavan et al., 2013). The plasma ChAT and BuChE activity also seem to be closely linked to each other to such a degree that e.g., the levels of ChAT protein in plasma are altered in subjects carrying a genetic variant of BuChE that has 30% reduction of the activity (Vijayaraghavan et al., 2013).

The CSF AChE activity did not differ between the three groups. Nonetheless, it correlated positively in all patients with the t-tau and p-tau levels. A similar finding was reported earlier (Johansson et al., 2013). AChE activity in CSF also correlated with CSF Aβ_42_ levels, but only in the MCI group. Currently, it is not known why the levels of AChE and tau protein correlate or what this association signifies. AChE is normally anchored within the synaptic cleft of the cholinergic neuronal interfaces. Given that increased tau levels in CSF may reflect ongoing synaptic and/or neuronal degeneration and given that cholinergic neurons are heavily affected by AD, the positive relationship between AChE and tau may reflect the ongoing synaptic degeneration in the brain.

The CSF *Cholinergic Index* was highest in patients at very mild stage of dementia. It also correlated positively with the Aβ_42_/p-tau ratio in the SCI group but negatively in the AD group. This may reflect the results of continuous ongoing insults on the cholinergic system in the brain, which predict inverse U-shape dynamic changes in the central cholinergic signaling in the course of dementia. This supports that changes in the central cholinergic system may play a crucial role in the underlying pathological events in dementia. Indeed, numerous pharmacoepidemiological reports suggest that undermining the proper cholinergic signaling by drugs with anti-cholinergic burden not merely induce symptoms of dementia but may in fact increase the future incidence of dementia (Jessen et al., 2010; Akter et al., 2015; Gomm et al., 2016; Chuang et al., 2017; Richardson et al., 2018; Coupland et al., 2019; Kumar A. et al., 2020; Kumar R. et al., 2020). Overall, these findings imply that treatment with ChEIs may be most effective if started early in asymptomatic patients as a preventive measure as is the case for cardiovascular disorders by treatment of high blood pressure or high cholesterol levels.

A limitation in this study is the known heterogeneity of the MCI and SCI groups which may have included pre-clinical AD patients. Nonetheless, samples were obtained from patients investigated according to clinical routine in a memory clinic, where they were clinically diagnosed as AD, MCI, or SCI. Another limitation is that the number of subjects was relatively small and thereby the study should be considered as a pilot study.

In conclusion, the present study showed that levels of both ChAT enzyme activity and ChAT protein in CSF and plasma samples differed between AD, MCI, and SCI patients. The finding that plasma levels of ChAT activity were significantly higher in AD than in SCI, whereas in CSF, the ChAT activity levels were higher in MCI patients, is interesting. This may indicate that ChAT activity is correlated with the progression of the disease. A decrease in CSF ChAT activity could predict the transition from MCI to AD. An opposite pattern was found in plasma, where increasing ChAT levels seem to follow the clinical pattern of the disease, i.e., from SCI to MCI to AD. This is particularly important since plasma sampling is a simple procedure compared to lumbar puncture to obtain CSF. However, our findings need to be confirmed in a larger cohort of AD and pre-clinical individuals. The inverse U-shape changes in the CSF *Cholinergic Index* suggested that cholinergic deficit can be regarded as a crucial player and not a bystander of the disease. This in turn emphasizes the need for research on whether early treatment with ChEIs represents an effective preventive measure against development of dementia disorders.

## Data Availability Statement

The original contributions presented in the study are included in the article/Supplementary Material, further inquiries can be directed to the corresponding author/s.

## Ethics Statement

The studies involving human participants were reviewed and approved by The Regional Ethics Committee in Stockholm County. The patients/participants provided their written informed consent to participate in this study.

## Author Contributions

AK performed the analyses and wrote the first draft. TD-S and AK revised the first draft and prepared the figures and tables. TD-S, MS, and ME revised the manuscript to the final draft for submission. All authors contributed to the article and approved the submitted version.

## Conflict of Interest

The authors declare that the research was conducted in the absence of any commercial or financial relationships that could be construed as a potential conflict of interest.

## Publisher’s Note

All claims expressed in this article are solely those of the authors and do not necessarily represent those of their affiliated organizations, or those of the publisher, the editors and the reviewers. Any product that may be evaluated in this article, or claim that may be made by its manufacturer, is not guaranteed or endorsed by the publisher.

## Supplementary Material

Supplementary Figure 1 Cerebrospinal fluid (CSF) ChAT activity in patients stratified based on cut-off levels of CSF AD biomarkers. Panel **(A)** shows CSF ChAT activity in patients with clinical diagnosis of AD, MCI, and SCI. The left panel represents cases who had CSF Aβ levels lower than the cut-off value of 650 pg/ml (Aβ+ group). The right panel represent cases with CSF Aβ_42_ levels greater than the cut-off value (Aβ− group). Panel **(B)** shows the corresponding stratification based on CSF total tau levels. The left panel represents cases with CSF t-tau levels greater than the cut-off value of 400 pg/ml (*Tau*+ group), while the right panel are cases with CSF t-tau levels lower than the cut-off value (*Tau*− group). NA = not available, indicating that none of the SCI patients had CSF t-tau levels greater than the cut-off value. Panel **(C)** shows the corresponding stratification based on CSF levels of phosphorylated tau (p-tau). The left panel represents cases with CSF p-tau levels greater than the cut-off value of 78 ng/ml (p-tau+ group), while the right panel are cases with CSF p-tau levels lower than the cut-off value (p-tau− group). These analyses should be considered with caution due to the small numbers of subjects. AD = Alzheimer’s disease; ChAT = choline acetyltransferase; CSF = cerebrospinal fluid; MCI = mild cognitive impairment; p-tau = phosphorylated tau181 protein; SCI = subjective cognitive impairment; t-tau = total tau protein. ^∗^*p* < 0.05, and ^∗∗^*p* < 0.01. The *p*-values are not adjusted for multiple comparisons.

Supplementary Figure 2 Cerebrospinal fluid (CSF)Cholinergic Index in patients stratified based on cut-off levels of CSF AD biomarkers. Panel **(A)** shows the CSF Cholinergic Index in patients with clinical diagnoses of AD, MCI, and SCI. The left panel represents cases who had CSF Aβ levels lower than the cut-off value of 650 pg/ml (Aβ+ group). The right panel represent cases with CSF Aβ42 levels greater than the cut-off value (Aβ− group). Panel **(B)** shows the corresponding stratification based on CSF total tau levels. The left panel represents cases with CSF t-tau levels greater than the cut-off value of 400 pg/ml (Tau+ group), while the right panel are cases with CSF t-tau levels lower than the cut-off value (Tau− group). NA = not available, indicating that none of the SCI patients had CSF t-tau levels greater than the cut-off value. Panel **(C)** shows the corresponding stratification based on CSF levels of phosphorylated tau (p-tau). The left panel represents cases with CSF p-tau levels greater than the cut-off value of 78 ng/ml (p-tau+ group), while the right panel are cases with CSF p-tau levels lower than the cut-off value (p-tau− group). These analyses should be considered with caution due to the small numbers of subjects. AD = Alzheimer’s disease; ChAT = choline acetyltransferase; CSF = cerebrospinal fluid; MCI = mild cognitive impairment; p-tau = phosphorylated tau181 protein; SCI = subjective cognitive impairment; t-tau = total tau protein. ^∗^*p* < 0.05, and #*p* < 0.09. The *p*-values are not adjusted for multiple comparisons. The Cholinergic Index was calculated as described in section “Materials and Methods.”
