# Supplementary figures and images for: CSF and Plasma Cholinergic Markers in Patients With Cognitive Impairment
Source: Front Aging Neurosci. 2021 Aug 26;13:704583. doi: 10.3389/fnagi.2021.704583 (PMC8426513; doi:10.3389/fnagi.2021.704583)

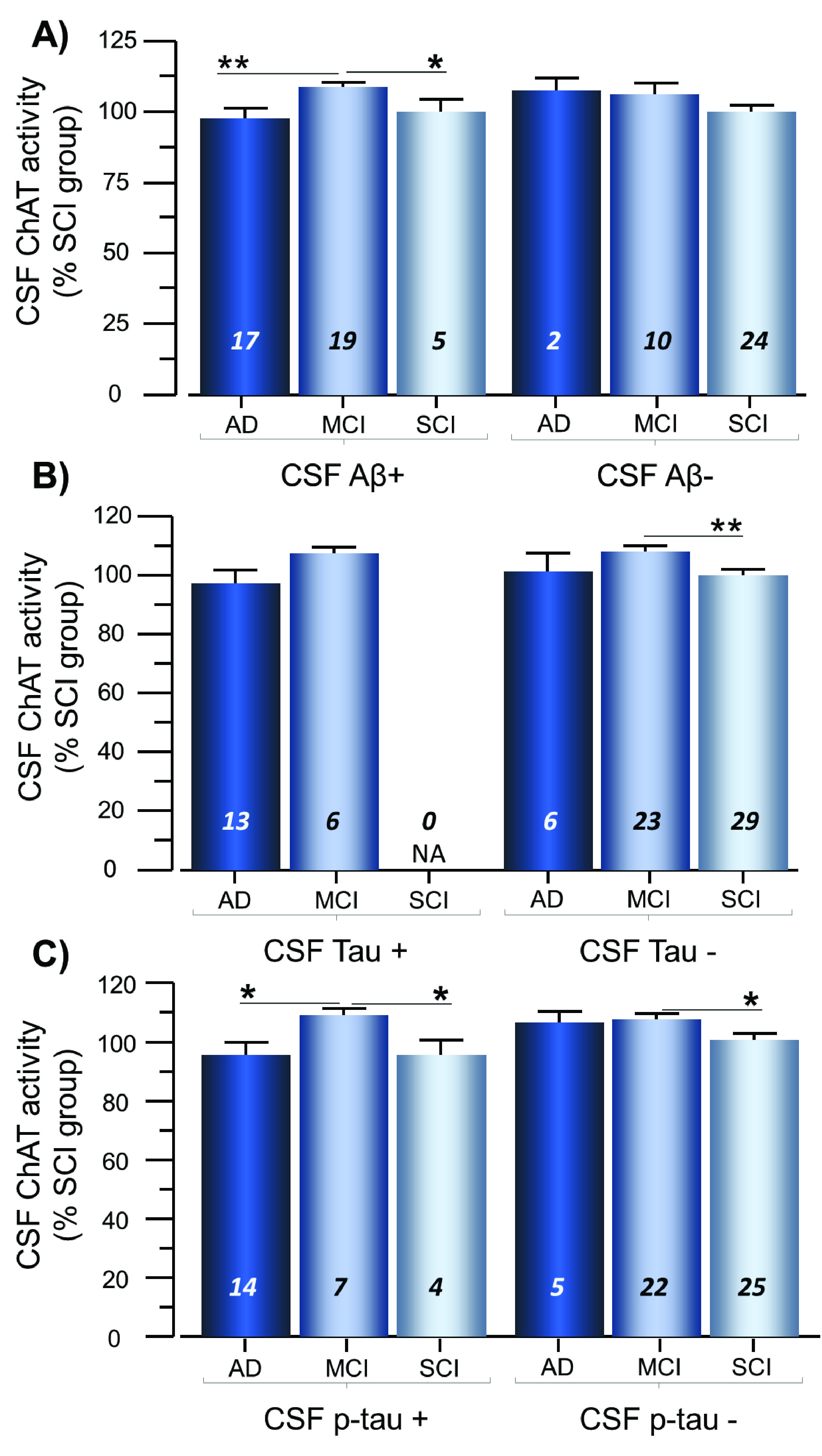

Supplement: Supplementary Figure 1 — Cerebrospinal fluid (CSF) ChAT activity in patients stratified based on cut-off levels of CSF AD biomarkers. Panel (A) shows CSF ChAT activity in patients with clinical diagnosis of AD, MCI, and SCI. The left panel represents cases who had CSF Aβ levels lower than the cut-off value of 650 pg/ml (Aβ+ group). The right panel represent cases with CSF Aβ42 levels greater than the cut-off value (Aβ− group). Panel (B) shows the corresponding stratification based on CSF total tau levels. The left panel represents cases with CSF t-tau levels greater than the cut-off value of 400 pg/ml (Tau+ group), while the right panel are cases with CSF t-tau levels lower than the cut-off value (Tau− group). NA = not available, indicating that none of the SCI patients had CSF t-tau levels greater than the cut-off value. Panel (C) shows the corresponding stratification based on CSF levels of phosphorylated tau (p-tau). The left panel represents cases with CSF p-tau levels greater than the cut-off value of 78 ng/ml (p-tau+ group), while the right panel are cases with CSF p-tau levels lower than the cut-off value (p-tau− group). These analyses should be considered with caution due to the small numbers of subjects. AD = Alzheimer’s disease; ChAT = choline acetyltransferase; CSF = cerebrospinal fluid; MCI = mild cognitive impairment; p-tau = phosphorylated tau181 protein; SCI = subjective cognitive impairment; t-tau = total tau protein. ∗p < 0.05, and ∗∗p < 0.01. The p-values are not adjusted for multiple comparisons. [file Image_1.JPEG]

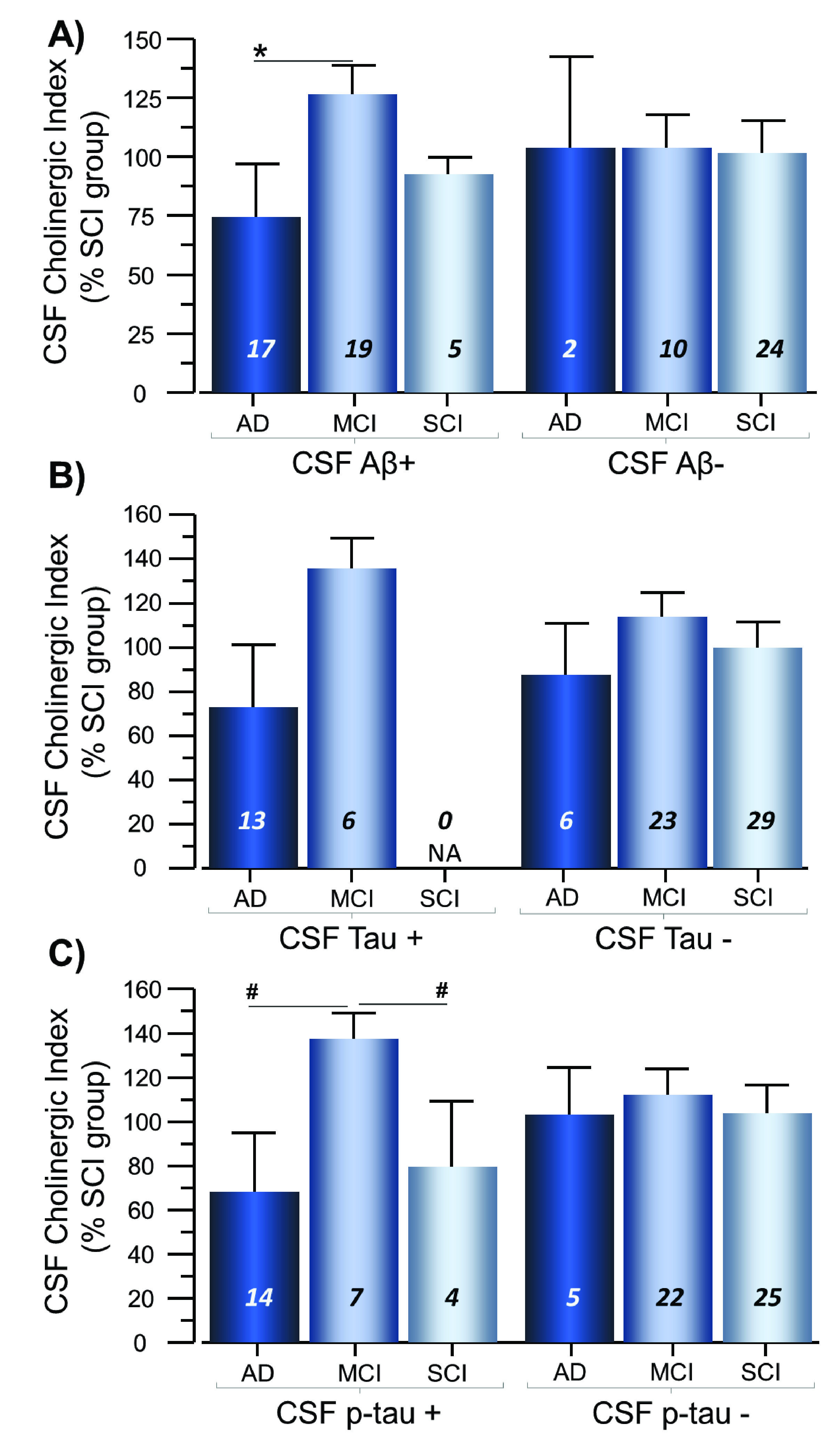

Supplement: Supplementary Figure 2 — Cerebrospinal fluid (CSF)Cholinergic Index in patients stratified based on cut-off levels of CSF AD biomarkers. Panel (A) shows the CSF Cholinergic Index in patients with clinical diagnoses of AD, MCI, and SCI. The left panel represents cases who had CSF Aβ levels lower than the cut-off value of 650 pg/ml (Aβ+ group). The right panel represent cases with CSF Aβ42 levels greater than the cut-off value (Aβ− group). Panel (B) shows the corresponding stratification based on CSF total tau levels. The left panel represents cases with CSF t-tau levels greater than the cut-off value of 400 pg/ml (Tau+ group), while the right panel are cases with CSF t-tau levels lower than the cut-off value (Tau− group). NA = not available, indicating that none of the SCI patients had CSF t-tau levels greater than the cut-off value. Panel (C) shows the corresponding stratification based on CSF levels of phosphorylated tau (p-tau). The left panel represents cases with CSF p-tau levels greater than the cut-off value of 78 ng/ml (p-tau+ group), while the right panel are cases with CSF p-tau levels lower than the cut-off value (p-tau− group). These analyses should be considered with caution due to the small numbers of subjects. AD = Alzheimer’s disease; ChAT = choline acetyltransferase; CSF = cerebrospinal fluid; MCI = mild cognitive impairment; p-tau = phosphorylated tau181 protein; SCI = subjective cognitive impairment; t-tau = total tau protein. ∗p < 0.05, and #p < 0.09. The p-values are not adjusted for multiple comparisons. The Cholinergic Index was calculated as described in section “Materials and Methods.” [file Image_2.jpg]
